# Impact of primary colorectal Cancer location on the *KRAS* status and its prognostic value

**DOI:** 10.1186/s12876-019-0965-5

**Published:** 2019-03-27

**Authors:** Ming-zhi Xie, Ji-lin Li, Zheng-min Cai, Ke-zhi Li, Bang-li Hu

**Affiliations:** Department of Research, Affiliated Tumor Hospital of Guangxi Medical University, 71 Hedi Road, Nanning, 530021 China

**Keywords:** Colorectal cancer, *KRAS* mutation, Tumor location, Prognosis

## Abstract

**Background:**

Colorectal cancer (CRC) originating from the right-sided or left-sided colon is distinct clinicopathological entity. The *KRAS* status and its prognostic value in CRC remain controversial. This study aimed to investigate the association of *KRAS* status with clinicopathological features and prognostic value in CRC.

**Methods:**

178 colon cancer and 145 rectal cancer patients were enrolled. *KRAS* mutation test was performed on paraffin-embedded tumor samples using PCR methods. The colon cancer was divided into right-sided colon cancer (RCC) and left-sided colon cancer (LCC). Studies that reported the association of *KRAS* mutation with CRC clinical features and prognosis in databases were searched prior to 2018. The data of the present study was combined with the data of published studies using meta-analysis methods.

**Results:**

No significant difference between colon cancer and rectal cancer regarding the *KRAS* status. The *KRAS* mutation was much frequent in RCC than in LCC (*p* = 0.010). 17 studies with 11,385 colon cancer patients were selected, the pooled results of our data and previous published data showed that *KRAS* mutation was more frequent in RCC compared with in LCC (*p* < 0.01); *KRAS* mutation was not associated with the prognosis in RCC patient; however, *KRAS* mutation indicated a poor prognosis in LCC patients compared with *KRAS* wild type (*p* < 0.01).

**Conclusion:**

*KRAS* status has no difference between colon cancer and rectal cancer. *KRAS* mutation was more frequent in RCC than in LCC, and associated with a poor prognosis in LCC patients, but not in RCC patients.

## Background

Colorectal cancer (CRC) is the third most common malignancy globally, accounting for approximately 10.0% of all new cancer cases [1]. CRC can be divided into colon cancer and rectal cancer base on their primary tumor location within the colon and rectum. The colon cancer can further be classified into right-sided colon cancer (RCC) and left-sided colon cancer (LCC) divided at the site of splenic flexure of colon. In recent years, a growing evidences revealed that there were significant differences between RCC and LCC with regard to the clinical findings, pathology, genetic mutations and survival time [2]. Thus, the location of tumor is an important factor that affects the prognosis of CRC.

Knowledge has shown that CRC tumorigenesis was characterized by the accumulation of genetic mutations, and Kirsten rat sarcoma viral oncogene homolog (*KRAS*) mutation was an early event in tumorigenesis [3]. *KRAS* mutation occur in approximately 30 to 50% of CRC, and 90% of mutation occur in codon 12 or 13 [4–6]. At present, anti-epidermal growth factor receptor (EGFR) antibody has been showed to be an effective therapy in the treatment of CRC patients. However, patients with *KRAS* mutation are unlikely to benefit from anti-EGFR therapy [7, 8], thus the *KRAS* status is used as an important biomarker for the selection of suitable patients. To date, many studies reported the clinicopathological features of CRC, and some studies further analyzed the *KRAS* status in RCC and LCC. However, the results of *KRAS* status in RCC and LCC remained inconsistent [9–11]. Here, we reported our results of clinicopathological features and *KRAS* status in Chinese patients with CRC, and further compared the *KRAS* status in RCC and LCC.

Although some studies showed that the tumor location and *KRAS* status could affect the effectiveness of patients treated with cetuximab [12, 13], the association between *KRAS* mutation and patients’ survival remained controversial, as some reports have failed to show any prognostic value of *KRAS* [14–17]. For example, there was a study found that *KRAS* mutations were not associated with risk of death in the overall patients of CRC, but LCC patients harboring *KRAS* mutation have a greater risk of death [18]. These results suggested that the prognostic value of *KRAS* status in CRC patients might also depending on the location of primary tumor; however, due to the limited of reports, this result still need to be further validated. Therefore, in this study, we combined the published data to further explore the prognostic value of *KRAS* status in both RCC and LCC.

## Methods

### Patients and data extraction

This study was retrospective design, patients who diagnosed with CRC and undergoing radical surgery in the Affiliated Tumor Hospital of Guangxi Medical University from January 2015 to January 2018 were included. The inclusion criteria were: CRC was confirmed by historical biopsy; Patients with inflammatory bowel disease or a known history of familial adenomatous polyposis were excluded. Patients with unknown *KRAS* status or receiving anti-EGFR agents in the perioperative period were also excluded. Detailed information was obtained on patients’ age, gender, histological differentiation, location of primary tumor, tumor infiltration, nodal status, distant metastasis, primary tumor American Joint Committee on Cancer (AJCC) stage. The location of primary tumor was determined by pathologic and operative reports. The right colon includes the cecum, ascending colon, liver flexure, and transverse colon, and the left colon includes the splenic flexure, descending colon and sigmoid colon. This study was approval by the Ethics Committee of Affiliated Tumor Hospital of Guangxi Medical University, written informed consent was obtained from each patient.

### DNA extraction from FFPE specimens

FFPE tumor blocks were selected by the surgical pathologist for clinical testing. Tissue was deparaffinized using xylene, ethanol washes, and acetone dehydration, and after cell lysis and proteinase K treatment, the DNA was extracted using the Puregene DNA Isolation or QIAquick PCR purification kit (QIAGEN, Inc. Valencia, CA).

### KRAS mutational analysis and sequencing

Mutations in *KRAS* codons 12 and 13 in exon 2 were detected using amplification refractory mutation system (ARMS)-PCR methods. KRAS mutation status was assessed with Human *KRAS* Gene 7 Mutations Fluorescence Polymerase Chain Reaction Diagnostic Kit (Amoy Diagnostics Co. Ltd., Xiamen, China) on the Agilent­Stratagene M × 3000P Q­PCR System (Agilent Technologies, Santa Clara, CA), according to the manufacturers’ instructions. The 7 most common *KRAS* mutations (p.G12D, p.G12 V, p.G12A, p.G12C, p.G12S, p.G12R, and p.G13D) in CRCs were detected. The reaction conditions included 1 cycle at 95 °C for 5 min; 15 cycles at 95 °C for 25 s, 64 °C for 20 s, 72 °C for 20 s; and a final 31 cycles at 93 °C for 25 s, 64 °C for 20 s, 72 °C for 20 s. Amplicons were detected using capillary electrophoresis on an ABI 3130xl Genetic Analyzer (Applied Biosystems/Life Technologies, Grand Island, NY) and analyzed using GeneMapper Software (Applied Biosystems/Life Technologies, Grand Island, NY).

### Search strategy for articles

Because the association of *KRAS* status with clinicopathological features and prognostic value in CRC might depend on the primary tumor location, we retrieval articles that analyzing the *KRAS* status and the prognostic value in RCC and LCC prior to April 2018 by searching the following electronic databases, PubMed, Cochrane Library, Web of Science, EBSCO and Chinese National Knowledge Infrastructure (CNKI). The following search terms were employed: “colon cancer”, “*KRAS*”, “left-side” or “right-side,” “prognosis”. Included articles were limited to human studies but not limited by language. The first author, year of publication, study location, number of *KRAS* status in LCC and RCC patients, hazard ratio (HR) and the corresponding 95%CI of prognostic value of *KRAS* status in LCC and RCC were extracted.

### Statistical analysis

Demographic and clinicopathological characteristics of the patients were stratified according to primary tumor location and *KRAS* mutation status. Continuous variables were presented as mean ± standard deviation, and compared using a Student’s t-test. Summary statistics for the patients were presented as totals for categorical variables. The differences between wild-type *KRAS* (wt- *KRAS*) and mutant-type *KRAS* (mt-*KRAS*) in each group were assessed by the χ^2^ test. The analyses were performed using R software version 3.4.3.

The meta-analysis of *KRAS* status in RCC and LCC, and the prognostic value of *KRAS* status in RCC and LCC was performed using Stata 11.2 software (Stata Corp, College Station, TX) with 2-tailed *p*-values. The pooled odds ratio (OR) with the corresponding 95% CI were used to estimate then *KRAS* status in RCC and LCC. The pooled HR with the corresponding 95% CI was used to assess the prognostic value of *KRAS* status in RCC and LCC. The *p*-value < 0.05 was considered statistically significant.

## Results

### Clinicopathological characteristics of CRC patients

There were 178 colon cancer and 145 rectal cancer patients finally enrolled in this study. The mean age of colon cancer and rectal cancer was (57.62 ± 12.67) years and (59.57 ± 11.89) years, respectively. No significant different between colon cancer and rectal cancer in the *KRAS* status (*p* = 0.393). Most of the colon cancer and rectal cancer was moderate differentiation, but no significant difference between colon cancer and rectal cancer (*p* = 0.099). There was no obvious difference in nodal status, distant metastases, AJCC stage between colon cancer and rectal cancer (*p* > 0.05). However, the number of advanced tumor infiltration patients (T3 + 4 stage) of colon cancer was greatly larger than that of rectal cancer (*p* = 0.018). Detail of included CRC patients was listed in Table 1.

**Table 1:** Characteristics of colon and rectal cancer patients

	Colon (*n* = 178)	Rectal (*n* = 145)	t/χ^2^ value	*P* value
Gender			0.209	0.648
Male	116	98		
Female	62	47		
Mean age	57.62 ± 12.67	59.57 ± 11.89	1.359	0.175
*KRAS* status			0.730	0.393
Mutation	62	44		
Wild-type	116	101		
Differentiation			4.617	0.099
Poor	34	22		
Moderate	137	122		
High	7	1		
Tumor infiltration			10.064	0.018
T1	2	8		
T2	19	27		
T3	32	21		
T4	125	89		
Nodal status			2.503	0.286
N0	62	63		
N1	57	40		
N2	59	42		
Distant metastases			3.388	0.066
M0	113	106		
M1	65	39		
AJCC stage			5.631	0.131
I	13	20		
II	41	36		
III	58	49		
IV	66	40		

### Clinicopathological characteristics of colon cancer in different status of KRAS

Of the 178 colon cancer patients, 62 occur *KRAS* mutation, the remained116 were *KRAS* wild-type. We divided these patients based on the status of *KRAS*, and found that no obvious differences in patients’ gender, age, histological differentiation, nodal status, distant metastases, AJCC stage (*p* > 0.05). However, significant difference was found between RCC and LCC regarding the *KRAS* status, with *KRAS* mutation in RCC was 46.4% (32/69), and in LCC was 27.5% (30/109), *p* = 0.010. Table 2 showed the detail of the characteristics in different status of *KRAS.*

**Table 2:** Characteristics of colon cancer in different *KRAS* status

	Mutation (*n* = 62)	Wild-type (*n* = 116)	t/χ^2^ value	*P* value
Gender			0.630	0.427
Male	38	78		
Female	24	38		
Mean age	56.63 ± 12.15	58.15 ± 12.94	0.761	0.448
Differentiation			0.218	0.897
Poor	12	22		
Moderate	47	90		
High	3	4		
Tumor infiltration			4.227	0.238
T1	1	1		
T2	5	14		
T3	7	25		
T4	49	76		
Nodal status			0.434	0.805
N0	22	40		
N1	18	39		
N2	22	37		
Distant metastases			2.029	0.154
M0	35	78		
M1	27	38		
AJCC stage			2.698	0.441
I	4	9		
II	12	29		
III	18	40		
IV	28	38		
Location			6.617	0.010
RCC	32	37		
LCC	30	79		

### Characteristics of included studies

**Fig. 1:**
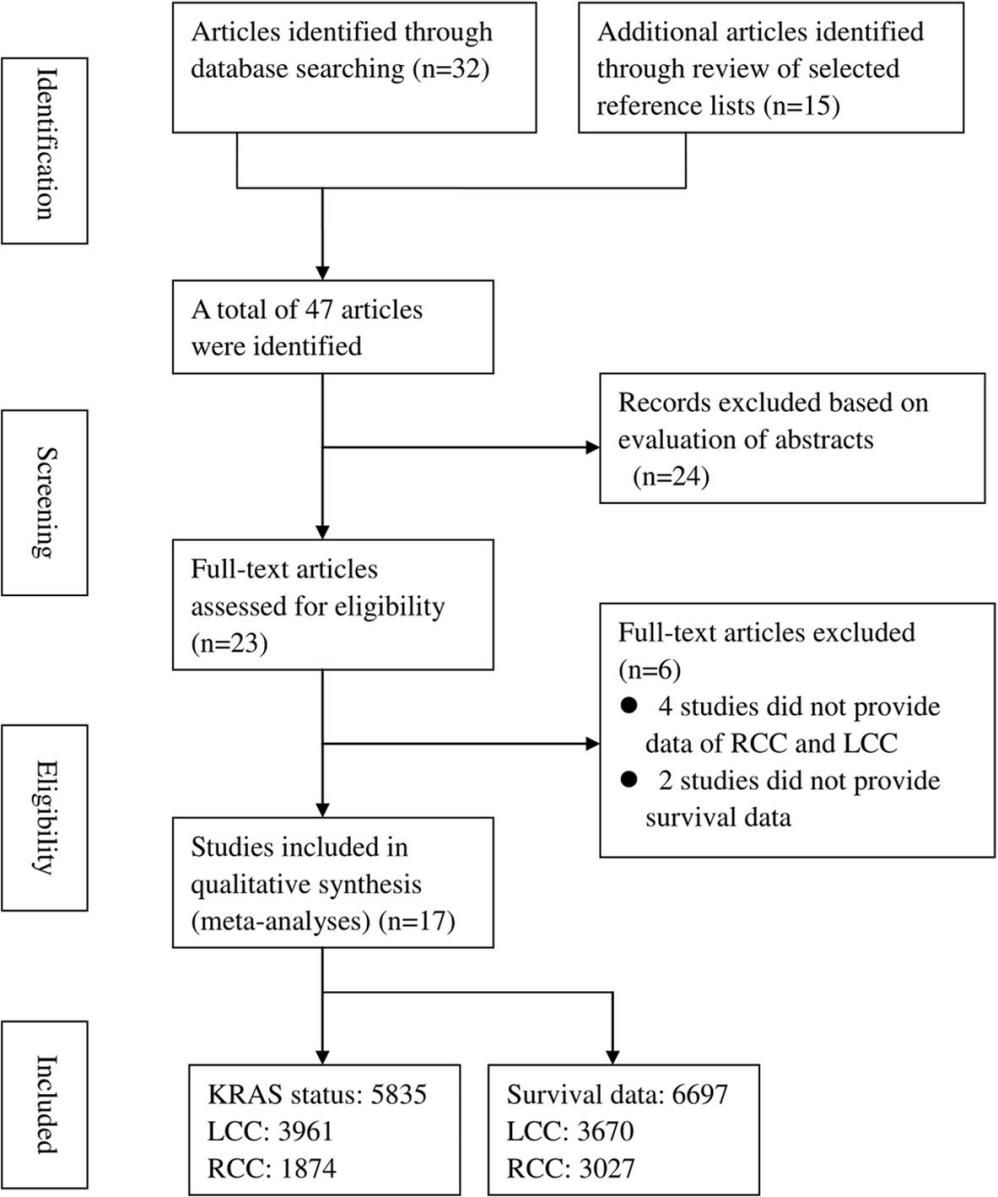
Flow diagram of the study selection process

Seventeen studies [9–13, 18–29] with 11,385 colon cancer patients were included in this study based on the included criteria. Among them, sixteen studies [9–13, 19–29] with 5, 835 patients provided the data of *KRAS* status in colon cancer, with 3961 LCC patients and 1874 RCC patients, respectively. Four studies [11, 18, 23, 28] with 6697 patients provided the survival data of *KRAS* status in colon cancer, with 3670 LCC patients and 3027 RCC patients, respectively. Table 3 presented the detail of the characteristics of included studies. A flow chart of the article selection process was shown in Fig. 1.

**Table 3:** Characteristics of included studies

Author	Year/country	Study design	LCC		RCC		LCC	RCC
			wt	mut	wt	mut	HR(95%CI)	HR(95%CI)
Chiu JW	2018/Canada	P	65	46	36	37	–	–
Hou Y	2018/China	R	196	117	62	86	–	–
Natsume S	2018/Japan	R	327	97	98	53	0.58 (0.37–0.86)	–
Charlton ME	2017/USA	R	2849		2701		1.18 (1.05–1.3)	0.93 (0.83–1.03)
Gao XH	2017/China	P	42	30	27	16	–	–
Kim ST	2017/Korea	R	115	9	25	4	–	–
Chang YY	2016/China	P	425	221	221	187	–	–
Sasaki K	2016/USA	R	201	96	65	64	1.81 (1.11–2.96)	1.03 (0.51–2.08)
Sun P	2016/China	R	60	48	44	40	–	–
Kodaz H	2015/Turkey	R	80	72	13	12	–	–
Ye JX	2015/China	R	100	50	94	67	–	–
Tong JH	2014/China	R	680	461	156	209	–	–
von Einem JC	2014/Germany	P	68	32	27	19	1.3 (0.68–2.34)	0.63 (0.43–0.92)
Cushman- Vokoun AM	2013/USA	R	13	16	21	15	–	–
Zhu XL	2012/China	R	79	45	59	47	–	–
Abubaker J	2009/Saudi Arabia	R	79	62	26	18	–	–
Bleeker WA	2000/Netherlands	R	26	3	16	10	–	–

*LCC* left-side colon cancer, *RCC* right-side colon cancer, *wt KRAS* wild type, *mut KRAS* mutant type, *HR* hazard ratio, *CI* confidence interval, *P* prospective, *R* retrospective

### Pooled results of KRAS status in RCC and LCC

**Fig. 2:**
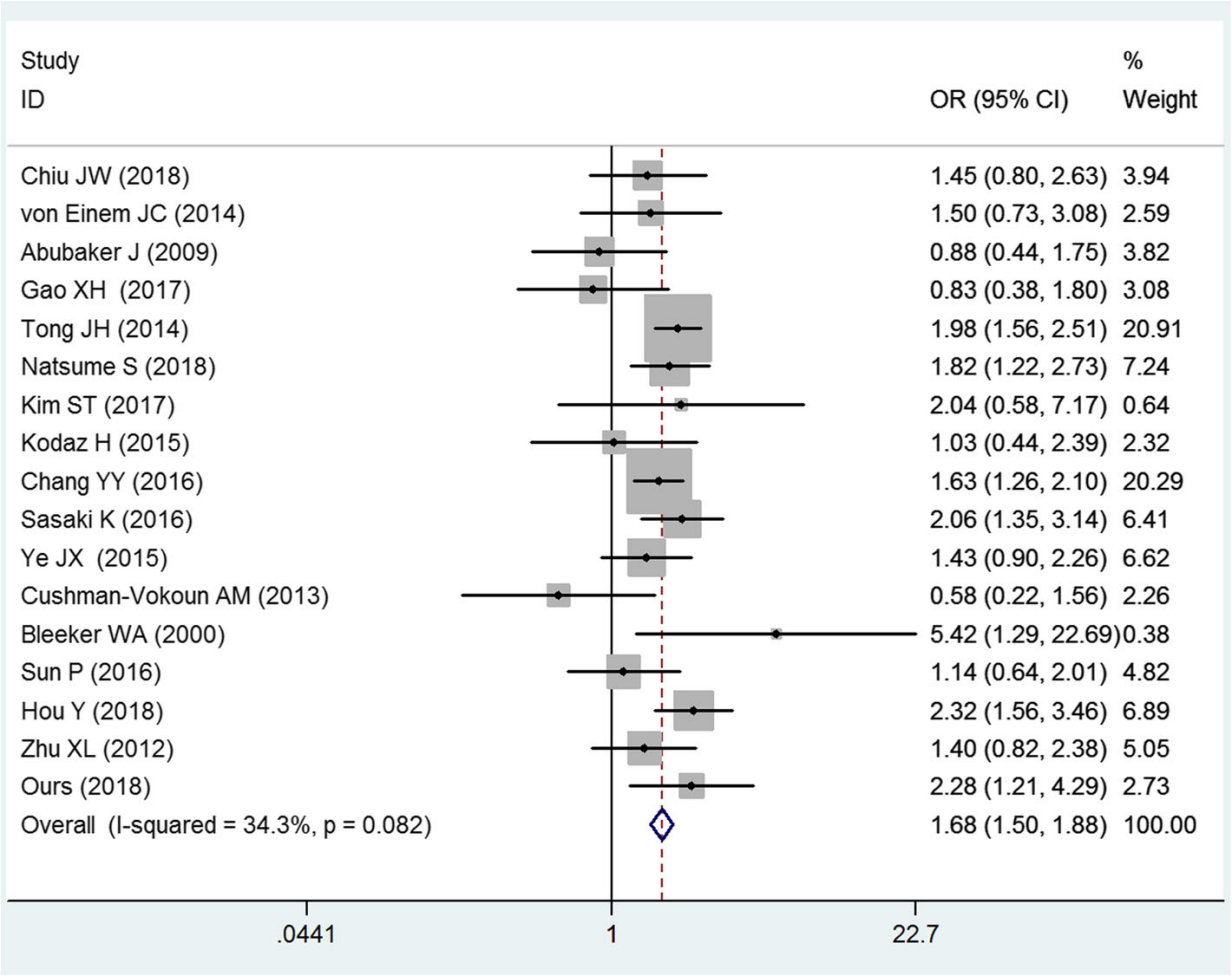
Forest plot of the *KRAS* mutation between right-sided colon cancer and left-sided colon cancer. The squares and horizontal lines correspond to the study-specific OR and 95% CI. The diamond represents the summary OR and 95% CI. The diamond locates to the right of vertical line means the *KRAS* mutation was much more frequent in RCC compared with in LCC (OR < 1)

**Fig. 3:**
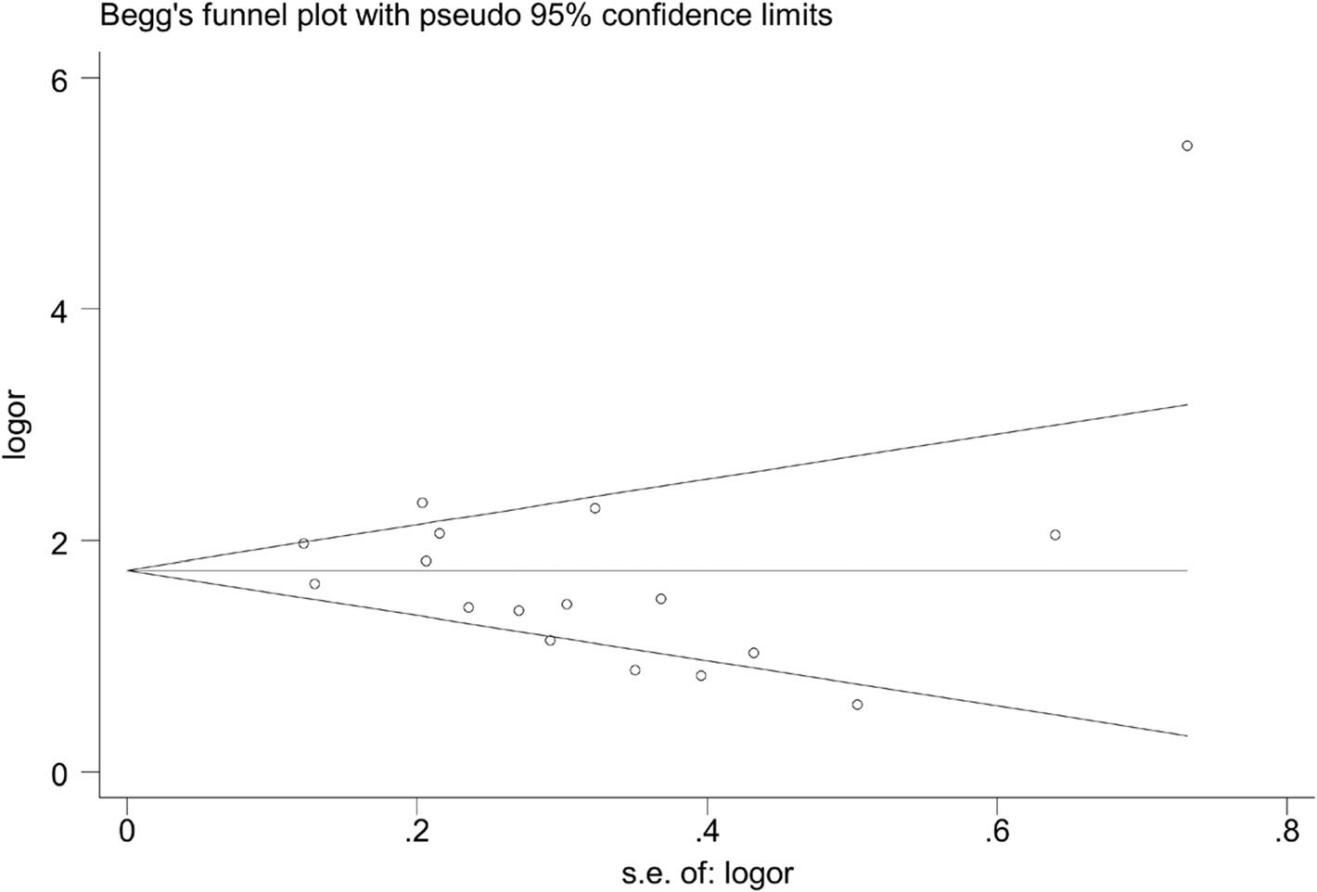
Begg’s funnel plot for publication bias test

To clarify the *KRAS* status in RCC and LCC, we combined the data from sixteen studies that provided the *KRAS* status in RCC and LCC with our data. The pooled results showed that *KRAS* mutation was much more frequent in RCC compared with LCC (OR = 1.68, 95%CI = 1.50–1.88, *p* < 0.01), and no significant heterogeneity across the studies (I^2^ = 34.3%, *p* = 0.082). See Fig. 2. There was little publication bias across the studies (Begg’s Test = 0.343; Egger’s test = 0.575). See Fig. 3.

### Pooled results of prognostic value of KRAS status in RCC and LCC

**Fig. 4:**
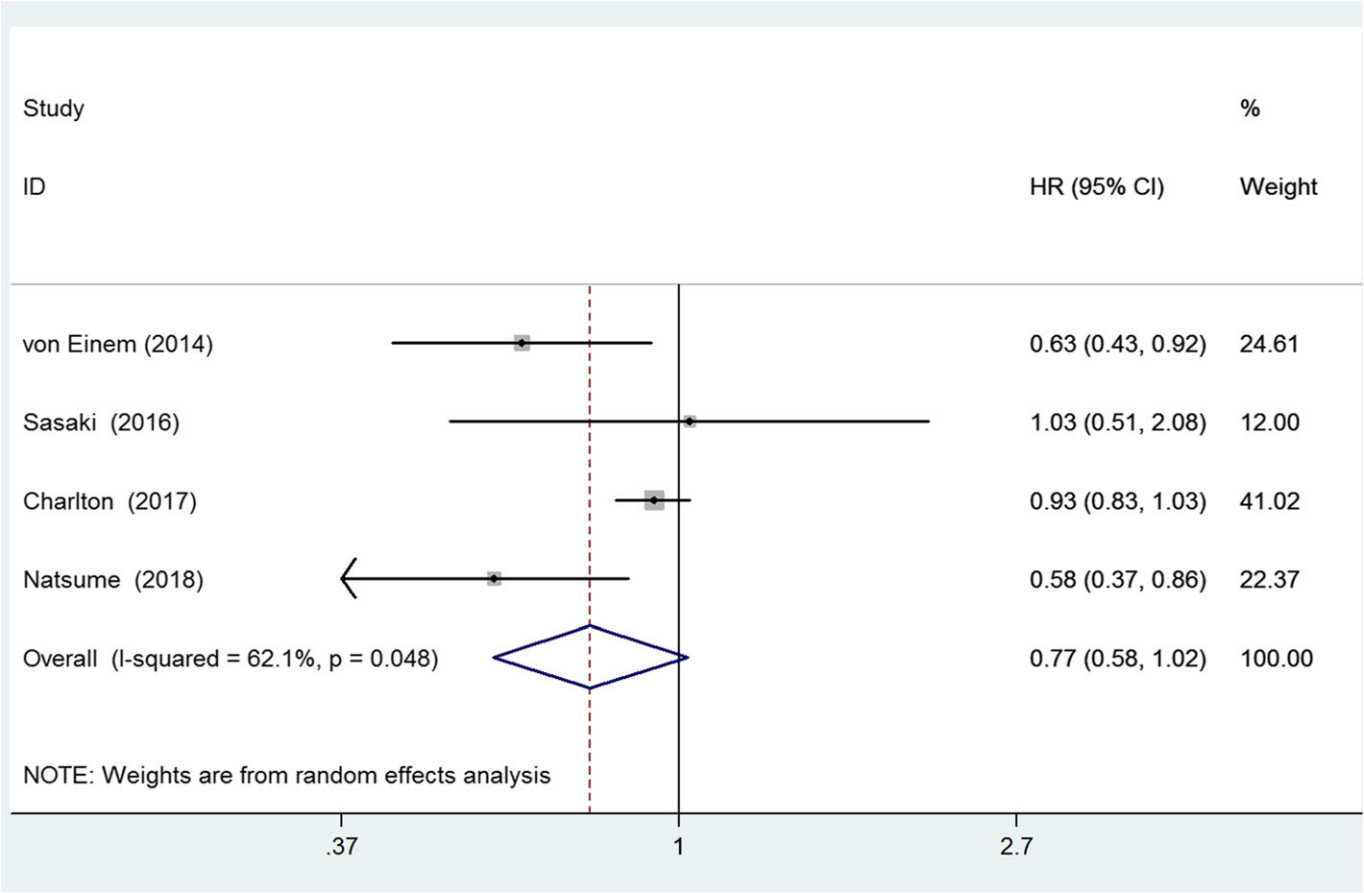
Forest plot of the *KRAS* mutation in the prediction of right-sided colon cancer patients. The squares and horizontal lines correspond to the study-specific HR and 95% CI. The diamond represents the summary HR and 95% CI. The diamond locates to the left but touches the vertical line means the no significant difference between patients with and without KRAS mutation regarding the OS

**Fig. 5:**
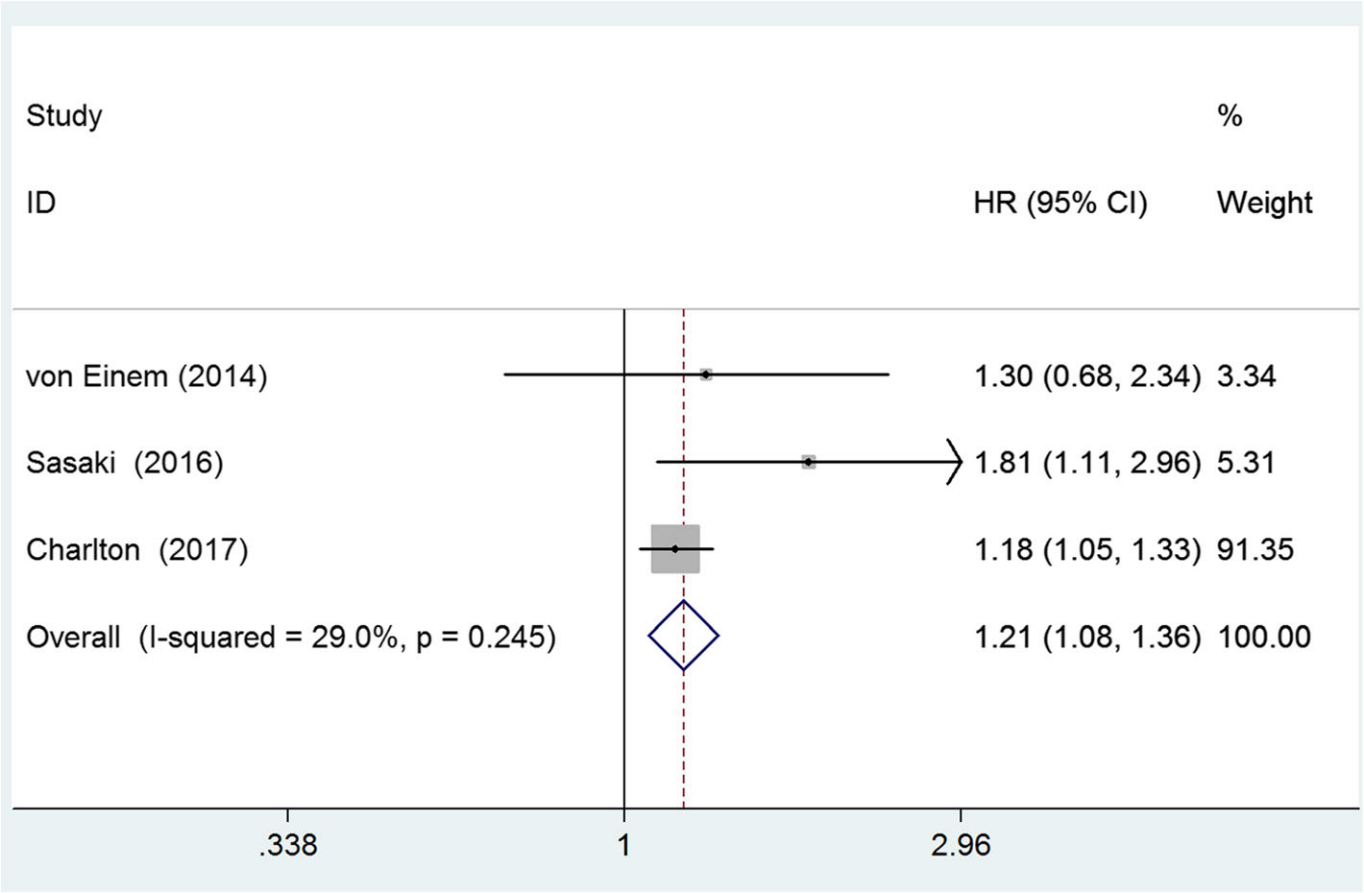
Forest plot of the *KRAS* mutation in the prediction of left-sided colon cancer patients. The squares and horizontal lines correspond to the study-specific HR and 95% CI. The diamond represents the summary HR and 95% CI. The diamond locates to the right of vertical line means the LCC patients with *KRAS* mutation has a shorter OS than patients with *KRAS* wild type (HR > 1)

To estimate the difference prognostic value of *KRAS* status in RCC and LCC, we combined the data from four studies that provided the data of overall survival (OS) of RCC and LCC patients with different *KRAS* status. All the studies included metastatic CRC cases, and patients receiving chemotherapy and/or radiotherapy after surgical resection. The multivariate analysis was performed by adjusting the confounding prognostic factors in each study. The pooled results showed that RCC patients with *KRAS* mutation has no significant different OS compared with patients with *KRAS* wild type (HR = 0.77, 95%CI = 0.58–1.02, *p* = 0.073; I^2^ = 62.1%), see Fig. 4; however, LCC patients with *KRAS* mutation has a shorter OS than patients with *KRAS* wild type (HR = 1.21, 95%CI = 1.08–1.36, *p* < 0.01; I^2^ = 29.0%), see Fig. 5.

## Discussion

In this study, by analyzing the clinicopathological features of 178 colon cancers and 145 rectal cancers, we failed to find the difference between colon cancer and rectal cancer regarding the *KRAS* status. By dividing the CRC based on the *KRAS* status, we did not observe the difference between *KRAS* mutation and wild type regarding the clinicopathological features, but found that RCC harboring more *KRAS* mutation compared with LCC (46.4% vs. 37.5%). We next combined the data of *KRAS* status in RCC and LCC, by pooling the data of sixteen studies and our data, we found that *KRAS* mutation was much more frequent in RCC than in LCC. In addition, by pooling the data of four studies, we found an obvious difference of OS in RCC and LCC regarding the *KRAS* status, that is, LCC patients with *KRAS* mutation has a shorter OS than with *KRAS* wild type, while RCC patients with *KRAS* mutation has no significant different OS compared with patients with *KRAS* wild type. These results indicated that both tumor location and *KRAS* status play important roles in the prognosis of CRC patients.

Knowledge has shown that the right and left sides of the colon have different embryologic origins. Tumor that origins from the two sites of the colon has different molecular carcinogenic characters, including *KRAS*, *BRAF* mutations and microsatellite instability (MSI) [12, 30, 31]. *KRAS* has been confirmed as a proto-oncogene which induces tumorigenesis in several cancers. In CRC, *KRAS* mutations status and tumor location are associated with targeted therapy effectiveness. In this study, *KRAS* status has no obvious difference in colon cancer or rectal cancer, but showed significant difference in RCC and LCC, which was in consistent with Natsume et al. [11] and Tong et al. [27] reports, but in contrast to Cushman-Vokoun et al. [10] report. Then the following meta-analysis with larger patients further verified the different *KRAS* status in RCC and LCC, indicating that the *KRAS* mutation was more frequent in RCC than LCC.

Since the effect of anti-EGFR therapy on CRC is associated with *KRAS* status, many studies have estimated the prognostic value of *KRAS* status in CRC patients [8–10], and some studies showed that mutation of *KRAS* indicated a poor prognosis of CRC patients, but there were also some reports have failed to show the similar result [14–17, 19], thus, the current conclusions regarding the prognostic value of *KRAS* status remain inconclusive. Because the distinct genetic alteration between RCC and LCC, both of the location of tumor and *KRAS* status are proposed to influence the prognostic value CRC. As Sasaki et al. [23] pointed that, *KRAS* mutation in RCC was not associated with the prognosis of CRC, while *KRAS* mutation in LCC indicated a poor prognosis of CRC patients. However, this result was based on a relative small sample size; thus, the robustness of the conclusion was undermined. In this study, by combining the data from four studies with 6697 patients, we found that LCC patients with *KRAS* mutation has a poor prognosis, but RCC patients with *KRAS* mutation did not show the similar results, which can partly explain the inconsistent results of the prognostic value of *KRAS* status in CRC patients. And these results further verified that both the *KRAS* status and location of tumor could affect the treatment effectiveness and prognosis in CRC patients.

Although this study showed the different *KRAS* status in RCC and LCC, and found the prognostic value of *KRAS* mutation was depending on the location of tumor, there were several limitations should be considered when interpreting the results. First, due to lack of the survival data in our center, we did not combine our data with published studies, thus, the sample size of the analysis for the prognostic value of *KRAS* status was relative small. Second, due to the limited studies available, we did not divide the patients based on the their ethnicity, so we did not know whether various ethnicity could affect the prognostic value of *KRAS* status in CRC, since evidence has shown that there were many differences in CRC between Asian and Caucasian ethnicity [32, 33]. Third, the present study only included the data of mutation of *KRAS* codons 12 and 13 in exon 2, other mutations, such as NRS and BRAF mutation, were not included. Although these type of mutations were fewer compared with the *KRAS* mutation, the lack of data of other mutations might lead to selected bias in the analysis. Fourth, the pooling analysis included all stages of CRC patients without stratified them into different stage, that is, early stage or advanced stage of CRC, hence the data very heterogeneous and would reduce the robustness of the results. Fifth, some of the included studies were retrospective design, which may lead selected bias and undermine the robustness of the results. Therefore, future research should be conducted to address the aforementioned limitations.

## Conclusion

This study demonstrated that no significant difference of *KRAS* status between colon cancer and rectal cancer. *KRAS* mutation was much more frequent in RCC compared with LCC, and LCC patients with *KRAS* mutation has a poor prognosis compared with *KRAS* wild type, but RCC patients did not show the similar effect.

## Abbreviation

CRC: Colorectal cancer
EGFR: Epidermal growth factor receptor
HR: Hazard ratio
*KRAS*: Kirsten rat sarcoma viral oncogene homolog
LCC: Left-sided colon cancer
OR: Odds ratio
RCC: Right-sided colon cancer
